# Supplementary material for: The genome sequence of the cryptorhynchine weevil, Kyklioacalles roboris (Stüben, 2003) (Coleoptera: Curculionidae)
Source: Wellcome Open Res. 2026 Jan 6;11:11. [Version 1] doi: 10.12688/wellcomeopenres.25416.1 (PMC12895102; doi:10.12688/wellcomeopenres.25416.1)
Supplement: Peer review history

# Reviewer report: Reviewer response for version 1

**DOI:** 10.21956/wellcomeopenres.28002.r145613

**Published:** 2026-02-11

**Authors:** João Pedro Marques, Juliana Sofia Alves

**Affiliations:** 1 BIOPOLIS Association - Research Network on Biodiversity and Evolution Biology (Ringgold ID: 637326), Vairão, Porto, Portugal; 2 University of Porto Faculty of Sciences (Ringgold ID: 131674), Porto, Porto District, Portugal

This manuscript presents a chromosome-level genome assembly for *Kyklioacalles roboris* generated within the Darwin Tree of Life framework. The study follows well-established and robust protocols, and the resulting assembly is of high technical quality, with strong contiguity, high consensus accuracy, and excellent BUSCO completeness. The data are clearly deposited and accessible, and the manuscript is generally well written and easy to follow.

 Overall, this genome represents a valuable resource for future evolutionary and comparative genomic studies of cryptorhynchine weevils and Curculionidae more broadly.

 However, I have a small number of comments that should be addressed to improve clarity, accuracy, and usability of the resource.

 General comments

 Although I recognise the intentionally concise nature of Darwin Tree of Life data notes, the manuscript would benefit from a brief element of biological interpretation. For example, it would be helpful to indicate whether the inferred number of pseudo-chromosomes is consistent with expectations for this species or closely related taxa, and whether the reported GC content is typical for cryptorhynchine weevils. In addition, a short statement highlighting potential future applications of this genome (e.g. comparative genomics, phylogenomics, or studies of cryptic diversification) would increase its value to readers.

 Specific comments

1. **Nucleic acid extraction**
  The authors state that an appropriate extraction protocol was selected based on sample characteristics. Please clarify which criteria were used to choose this protocol.
2. **Figure 1**
  Please consider adding a physical scale or size reference to the specimen photograph.
3. **Assembly curation section**
  It is not explicit whether any duplication or haplotypic purging step was applied. If purging was handled implicitly by hifiasm (e.g. through default or optional parameters), this should be clearly stated, as haplotypic purging is a standard step in current assembly workflows.
4. **Table 2**
  The line "Longest scaffold length (Mb)" contains formatting issues that should be corrected.
5. **Figure 3 (Hi-C contact map)**
  Chromosome 18 appears to be missing from the top legend. Please update the figure accordingly.
6. **Table 4**
  The entry “Total BUSCO groups searched (2124)” is incorrectly formatted and should be corrected.
7. **Figure 6**
  The Y-axis label appears to be inverted or incorrectly oriented. Please check and correct.

Are sufficient details of methods and materials provided to allow replication by others?

Yes

Is the rationale for creating the dataset(s) clearly described?

Yes

Are the datasets clearly presented in a useable and accessible format?

Yes

Are the protocols appropriate and is the work technically sound?

Yes

Reviewer Expertise:

Evolutionary Biology, Population Genomics, Bioinformatics

We confirm that we have read this submission and believe that we have an appropriate level of expertise to confirm that it is of an acceptable scientific standard.

# Reviewer report: Reviewer response for version 1

**DOI:** 10.21956/wellcomeopenres.28002.r144321

**Published:** 2026-01-28

**Authors:** Nikoletta Andrea Nagy

**Affiliations:** 1 University of Debrecen, Debrecen, Hungary

The manuscript is well written, and the genome presented is of high quality and can contribute to future research on weevils. I have only a few suggestions for the authors.

 Background

 “In Britain it is the least often encountered of the three similar species in this group” – please list the other two species

 Figure 1

 Please put a scale on the photo

 Assembly statistics

 I would recommend including whether the assembled mitochondrial genome is an incomplete linear or a complete circular sequence.

 Table 2

 Longest scaffold length – the “-“ character should be placed in the “icKykRobo1.hap2.1” column

Are sufficient details of methods and materials provided to allow replication by others?

Yes

Is the rationale for creating the dataset(s) clearly described?

Yes

Are the datasets clearly presented in a useable and accessible format?

Yes

Are the protocols appropriate and is the work technically sound?

Yes

Reviewer Expertise:

arthropod genomics, transcriptomics, behavioural genetics

I confirm that I have read this submission and believe that I have an appropriate level of expertise to confirm that it is of an acceptable scientific standard.
